# On the Treatment of Empyema by Chassagnac’s Drainage Tube

**Published:** 1864-04

**Authors:** W. D. Slater

**Affiliations:** Chicago; Graduate of Royal College of Physicians of England; Royal College of Surgeons of London and Dublin; late House-Surgeon Westminster Hospital, London


### ON THE TREATMENT OF EMPYEMA BY CHASSIGNAC'S DRAINAGE TUBE.

By W. D. SLATER, M. D., of Chicago;

*Graduate of Royal College of Physicians of England; Royal College of Surgeons of London and Dublin; late House-Surgeon Westminster Hospital, London.*

Empyema is, perhaps, one of the most fatal and distressing diseases which comes under the notice of the Physician, and one in which every plan of treatment too often fails to afford any permanent relief, consequently any treatment which may restore the patient to life as it were, and in many cases be the means of curing the disease is well worth the earnest consideration of the profession.

Some time since, M. Chassignac of Paris, undertook to treat Chronic Abscesses by means of what is called the India Rubber Drainage Tube, consisting of a long india rubber tube which had been pierced at short intervals by numerous small holes, large enough, however, to allow the pus freely to escape. The tube was passed completely through the Abscess in the same manner as an ordinary seton thread, and the ends tied together so as to prevent any air making its way into the cavity of the Abscess. This treatment was followed with such marked success, that it was determined to try it in cases of Empyema requiring operative interference, and the results, so far, have been very encouraging.

The simplest manner in which the tube is introduced is as follows :—A trochar and canula having been passed into the pleura from behind, and the trochar withdrawn, a long probe to which the tube has been tied, is passed through the canula and forwards across the cavity of the pleura, and brought out between the ribs in front, this then cut down upon and withdrawn, together with the end of the tube. The canula is then taken out, and the ends of the tube tied together. Pus immediately commences to escape through the small openings in the tube and the air is effectually excluded.

The operation itself is simple and gives very little pain; the inconvenience and irritation from wearing the tube is slight and does not require any interference whatever. The exact place in which the operation should be performed depends altogether on circumstances, and the operator should be guided by the rules laid down for paracentesis thoracis.

The advantage of this treatment is, that by the constant draining off of the pus, and its not being allowed to again collect in the pleura in any quantity, the lung is allowed to expand and remain expanded, thereby relieving the patient very materially, and at the same time giving other remedies and general treatment a much better chance of success than by the ordinary method of tapping.

In conclusion I give the history of two cases, in which this operation was performed, during the term of my House-Surgency at the Westminster Hospital.

Wm. Day—aged 22, sailor, admitted into Westminster Hospital, December, 1861. It was stated by his friends that a short time previously, he had suffered from pleurisy of the left side. A medical man had been called in but as he continued to get worse daily he was brought to the Hospital.

On examination, the left side was found to be distended with fluid, great dullness on percussion, no respiratory murmur to be heard, great difficulty of respiration, puerile breathing on right side, heart pushed a little to the right side, action labored, sounds normal, pulse very small and weak, countenance pale, anxious, and distressed, and the patient was altogether very weak.

The usual remedies to promote absorption were immediately employed and plenty of stimulants given, but in vain, for he continued to get worse for three days, when, owing to the exhausted state of the patient and the extreme dyspnœa, it was decided to tap the pleura, which was done on the fourth day from his admission. An exploring needle having been first introduced, nearly two pints of clear, healthy pus were drawn off. This operation relieved him very much, but in a few days the sac commenced to refill, when it was determined to try the drainage plan, which was accordingly done. The pus escaped freely through the openings in the tube; he was placed on a nourishing and stimulating diet; blisters and other remedies were employed. He continued rapidly to recover; the pus gradually ceased to flow, and in six weeks from the time of its introduction, the tube was withdrawn. In two weeks from that date he was discharged perfectly cured.

James Barrington—aged 36, engineer, admitted into Hospital May, 1861. Has been suffering from pleurisy, for which he had been attended, but becoming much worse, his friends brought him to the Hospital.

On admission he was found to be suffering from a considerable effusion into the left pleural sac. Nearly three-fourths of the lower part of the chest was dull on percussion and the respiratory murmur was inaudible, the upper third was clear, and a distinct respiratory murmur could be heard, breathing on the right side was puerile, heart sounds healthy, pulse very weak, countenance distressed and anxious, dyspnœa very great.

The usual remedies were employed, but as he did not get any better during the week, it was decided to try the drainage tube. This operation was performed.

For the first twelve hours, he remained in pretty much the same condition, but after that time, continued steadily but slowly to improve.

He was allowed plenty of stimulants and a nourishing diet, together with the usual remedies. After wearing the tube nearly five weeks, it was withdrawn, and between three and four weeks after, he was discharged cured.

